# Impact of educational attainment and economic globalization on obesity in adult females and males: Empirical evidence from BRICS economies

**DOI:** 10.3389/fpubh.2023.1102359

**Published:** 2023-02-14

**Authors:** Gamze Sart, Yilmaz Bayar, Marina Danilina

**Affiliations:** ^1^Department of Educational Sciences, Hasan Ali Yucel Faculty of Education, Istanbul University-Cerrahpaşa, Istanbul, Turkey; ^2^Department of Public Finance, Faculty of Economics and Administrative Sciences, Bandirma Onyedi Eylul University, Bandirma-Balikesir, Turkey; ^3^Department of Economics, Plekhanov Russian University of Economics (PRUE), Moscow, Russia; ^4^Department of Economics, Financial University Under the Government of the Russian Federation, Moscow, Russia

**Keywords:** education, economic globalization, obesity, causality analysis, cointegration analysis

## Abstract

Obesity has considerably increased since 1980 and become a global epidemic. Obesity-related health problems and the negative social and economic implications of obesity have led international institutions and countries to combat it. This study investigates the role of educational attainment and economic globalization in the global prevalence of obesity in samples of adult females and males in BRICS economies for 1990–2016 through causality and cointegration tests. The results of the causality tests reveal that educational attainment and economic globalization have a significant influence on obesity in both adult females and males in the short run. Furthermore, cointegration analysis indicates a negative effect of educational attainment on obesity in all BRICS economies in the long run, but the influence of economic globalization on obesity differs among the BRICS economies. Furthermore, the negative influence of educational attainment on obesity is revealed to be relatively higher in females than males.

## 1. Introduction

Obesity has become a serious public health and economic problem in the globalized world, and the World Health Organization (WHO) accepted obesity as a global epidemic in 1997 (1). On the one hand, obesity can be a significant threat to public health in terms of life expectancy, life quality, and being the source of many non-communicable diseases (NCDs) such as cancer, type 2 diabetes, coronary heart disease, and stroke (2). On the other hand, obesity can negatively influence economies by decreasing life expectancy and productivity and increasing health care expenditures and disability (3). However, the prevalence rates of obesity are continuing to increase in all countries. The World Obesity Federation (WOF) predicts that one in five women and one in seven men will become obese [in other words, their body mass index (BMI) will be greater than or equal to 30 kg/m^2^], and, in turn, over a billion people worldwide will be obese (4). So, obesity is more prevalent in women than men and this trend is predicted not to change in the near future. Furthermore, most obese people have been living in low- and middle-income countries (LMICs), and the number of obese people in LMICs and low-income countries has doubled and more than tripled, respectively, as of 2010 (4).

Countries and international institutions, such as the WHO and the United Nations, have tried to combat obesity given its negative health and economic effects. In this context, the WHO's Global Strategy on Diet, Physical Activity and Health suggests actions to support healthy diets and regular physical activity and calls for stakeholders to take action at local, regional, and global levels to improve the diets and physical activity patterns of individuals (5). Furthermore, the “Global Action Plan on Physical Activity 2018–2030: More Active People for a Healthier World” by the WHO makes policy suggestions to raise physical activity (6). Obesity is not explicitly mentioned in the 17 Sustainable Development Goals (SDGs), but it is implicitly targeted in the context of SDG-2 (zero hunger), SDG-3 (good health and wellbeing), and SDG-12 (responsible consumption and production) (7).

The unveiling of the factors underlying obesity are critical for policy-making to combat it. In this context, economic development, technological progress, dietary factors, physical activity, sleep duration, genetics, demographics, social and lifestyle factors, stress levels, environment, and built environment have been documented as the major factors underlying obesity (8–14). However, the determinants of obesity vary considerably between countries based on their economic and social development levels. In this research, the influence of educational attainment and economic globalization on obesity is separately investigated in adult females and males because educational attainment can also influence most of the factors underlying obesity, and the economic globalization can also affect obesity through facilitating the flows of capital, goods and services among the countries. Furthermore, obesity is more prevalent in females than males in accordance with global obesity distribution by gender (4). In this context, the means of obesity in adult females and males in the BRICS economies are respectively 16.562% and 7.768 during the 1990–2016 period and consistent with World Obesity Federation (4).

Educational attainment is expected to influence obesity through the following channels: (a) education is a significant factor underlying economic growth and development; (b) education is a significant factor underlying personal income and life quality; (c) individuals with higher education are more aware of the determinants of obesity and the associated health risks; (d) individuals with higher education have greater access to information about healthy living and healthcare services (15). Hence, Cutler and Lleras-Muney (16) discovered that individuals with higher education levels are less likely to be obese, smoke, drink a lot, or use illegal drugs. Therefore, a negative influence of education on obesity is expected, depending on countries' economic development levels.

On the other hand, the world has experienced a significant globalization process as of 1980, and, in turn, the mobility of goods, services, and individuals has considerably grown, and economies and societies have integrated to a great extent. As a result, economic globalization has led to many economic and non-economic changes in the world. In this context, economic globalization can influence obesity in different ways through diverse channels: (a) economic globalization can affect obesity through economic growth and development; (b) economic globalization can ease the entry of food manufacturers and supermarket and fast-food chains into countries and, in turn, foster obesity by increasing accessibility to obesogenic products; (c) economic globalization can influence obesity through the dissemination of the modern workplace, technology use, and motorized transportation; (d) economic globalization can affect obesity through urbanization and cultural changes (17, 18). Therefore, the influence of economic globalization on obesity can change depending on which factors are dominant in the relationship between economic globalization and obesity.

Furthermore, there is a close interaction between educational attainment and globalization. Education is also internationalized and new concepts such as knowledge economy and lifelong learning are integrated with education policies (19). The countries have increased their education investments and updated their education curriculum and teaching methods to survive in the highly competitive global economy. The globalized world has also experienced the significant technological progress during the past four decades and in turn the need for a highly skilled workforce is increased in the global labor markets. As a result, educational attainment is going to increase in the world through demand and supply side causes such as higher income and the need for a highly skilled workforce (20) and thus economic globalization can also affect the obesity through the channel of education attainment.

Extensive empirical studies have been conducted on the determinants of obesity in different samples from various countries. This research aims to make a contribution to the literature about the determinants of obesity in three ways. First, the study is one of the first studies to investigate the interaction among educational attainment, economic globalization, and obesity in samples of the economies of Brazil, Russia, India, China, and South Africa (BRICS). BRICS economies are the drivers of global economic expansion and account for 40% of world population, 25% of nominal global GDP, and 30% of world land coverage, and 18% of international trade (21). Second, the influence of educational attainment and economic globalization on obesity has been relatively less explored, and studies have generally utilized the regression approach and the regression analysis enables us to see the common effect of a variable on dependent variable for all countries. Therefore, another novelty of the study is the utilization of causality and cointegration tests to determine the short- and long-term influence of educational attainment and economic globalization on obesity for each country in the sample. Finally, the study investigates the interaction of educational attainment, economic globalization, and obesity through macro-data, unlike many empirical studies, and its findings may be useful for policy-making to combat obesity. The next part of the paper evaluates and summarizes the empirical studies in the relevant literature, and the data and methods are explained in Section 3. The econometric applications are conducted and their findings are evaluated in view of the related literature in Section 4. The paper comes to its conclusion in Section 5.

## 2. Literature review

Obesity is a global epidemic and the source of many diseases and social and economic problems. Therefore, the determinants of obesity have been explored in a widespread manner; economic development, technological progress, dietary factors, physical activity, sleep duration, genetics, demographics, social and lifestyle factors, stress levels, environment, and built environment have been documented as the major factors underlying obesity (8–14). However, the influence of educational attainment and economic globalization, which also affect all these factors, on obesity has not been explored sufficiently. The influence of educational attainment, including nutritional, physical, and virtual education, on obesity has been investigated relatively more when compared with economic globalization.

The literature summary on the various education indicators–obesity nexus in Table 1 shows that researchers have generally utilized regression analysis and reached the conclusion that education proxied by different indicators has generally had a negative influence on obesity in countries with diverse economic development levels (15, 23–30). However, the interaction between lower education and obesity was generally weaker in men than women (22, 29). Monteiro et al. (22) also found that education did not have a significant impact on obesity risk in men in the less-developed region of Brazil. Furthermore, Curry (12) discovered an insignificant influence of educational attainment on risk of being obese among Black women in the United States. The literature research has uncovered that the influence of educational attainment and economic globalization on obesity has not been analyzed in sample of BRICS economies yet. Therefore, this study investigated the interaction among obesity, educational attainment, and economic globalization in sample of the BRICS economies, the drivers of the global economy during the past a few decades.

**Table 1:** Literature summary on the education–obesity nexus.

**Study**	**Sample**	**Method**	**Impact of education on obesity**
Monteiro et al. (22)	Brazil	Logistic regression	Education did not have a significant impact on obesity risk for men in the less-developed region. But education had a negative impact on obesity risk for men in the more-developed region. There existed a negative interaction between education and obesity risk for women in both regions
Anyanwu et al. (23)	Nigeria (325 males and 254 females with Ibo ethnicity)	Descriptive statistics and correlation analysis	Obesity was the highest in the group with the lowest education
Devaux et al. (15)	Australia, Canada, England, and Korea	Regression analysis	Negative
Faeh et al. (24)	Switzerland (53,588 adult individuals)	Logistic regression	Negative
Brunello et al. (25)	Austria, Denmark, Germany, Greece, Italy, Portugal, Spain, Sweden, and the United Kingdom	Regression analysis	Negative
Chung et al. (26)	Republic of Korea	Logistic regression	Negative in both adult men and women
Chung and Lim (27)	Republic of Korea (14,577 women)	Extended Oaxaca–Blinder method	Obesity was much more prevalent in women with less education, and lifestyle factors contributed most to obesity
Curry (12)	Black women in the United States	Regression analysis	Insignificant
Hsieh et al. (28)	Taiwan (28,092 old men and 31,835 old women)	Logistic regression	Negative
Witkam et al. (29)	Meta-analysis about the studies on the nexus of education and obesity		Negative, but the interaction between lower education and obesity was weaker in men than women
Iriyani et al. (30)	Indonesia (38 adult participants from Samarinda City Junior High School)	Quasi-experiment approach	Nutritional education and physical activity contributed to weight loss
Milla et al. (31)	Indonesia (19 participants from Surabaya)	Quasi-experimental approach	Virtual education had a positive influence on obesity awareness

**Source:** Authors own elaboration based on literature research.

The influence of globalization and its main dimensions on obesity has been investigated by very few researchers in Table 2, and they have generally utilized regression analysis to find a positive influence of globalization on obesity (17, 18, 32–34, 36–38). However, Ghosh (35) found that the influence of globalization on obesity changed depending on countries' income levels.

**Table 2:** Literature summary on the globalization–obesity nexus.

**Study**	**Sample**	**Method**	**Impact of globalization on obesity**
De Vogli et al. (32)	127 countries	Regression analysis	Economic globalization had a positive influence on obesity
Goryakin et al. (33)	56 countries (887,000 women)	Regression analysis	Positive
Costa-Font and Mas (34)	26 countries	Regression	Positive
Ghosh (35)	Asian countries	Westerlund cointegration test	Economic and social globalization had a positive influence on obesity in low- and low-middle income countries, but globalization had a negative influence on obesity in relatively richer economies
Lopez et al. (36)	44 low- and middle-income economies	Regression analysis	Trade liberalization made a positive contribution to the spread of sugar-sweetened beverages
Lin et al. (37)	172 countries	Regression analysis	Sugar and processed food imports had a positive influence on obesity
Fox et al. (38)	190 countries	Regression analysis	Economic globalization had a positive influence on obesity, but the influence of cultural globalization on obesity varied among the countries
García (17)	10 Latin American and Caribbean economies (320,873 non-pregnant women)	Multiple logistic regression	Positive
An et al. (18)	Review of 16 studies about the globalization–obesity nexus		14 studies revealed a positive interaction between economic globalization and obesity, one study revealed a negative interaction between two variables, and one discovered an insignificant interaction between two variables

**Source:** Authors own elaboration based on literature research.

## 3. Data and method

This article studies the effects of educational attainment and economic globalization on obesity in females and males in BRICS economies for 1990–2016 through cointegration and causality tests. In the econometric analyses, adult obesity (OBS) is proxied by males or females with BMIs of 30 kg/m^2^ or higher as a percentage of the male/female population aged 18+ and is obtained from the World Bank database (39). The BMI is calculated through weight (kilograms) divided by squares of the height (meters). Educational attainment (EDU) is substituted by the mean years of schooling of males/females by UNDP (40), and economic globalization (EG) is substituted with the economic globalization index calculated by the KOF Swiss Economic Institute (41) and measures the trade and financial globalization and gets value between 1 and 100 (higher values reflect higher economic globalization level). All series are yearly, and the study period is specified as 1990–2016 because adult obesity data is available for this period.

The main characteristics of the obesity, educational attainment, and economic globalization reported in Table 3 indicate that the means of obesity in adult females and males are, respectively, 16.562% and 7.768, so obesity is more prevalent in females than males in the BRICS economies. Furthermore, South Africa, Russia, and Brazil had a larger obesity rate than China and India, and females also had considerably larger obesity rates in these countries than males. On the other hand, the mean years of schooling are 6.93 years in females and 7.63 years in men, and the gap in schooling years by gender is relatively very low. However, females had relatively larger schooling years in Brazil and Russia, but males had relatively larger schooling years in China, India, and South Africa. The mean economic globalization level is 41.203 in BRICS economies during 1990–2016 and Russia and South Africa had relatively higher economic globalization level. Furthermore, variations in obesity and economic globalization levels in these countries are larger than those in education.

**Table 3:** Main characteristics of the variables.

**Characteristics**		**Females**			**Males**		
		**OBS** ^*^	**EDU** ^**^	**EG** ^***^	**OBS** ^*^	**EDU** ^**^	**EG** ^***^
Mean		16.562	6.933	41.203	7.768	7.631	41.203
Maximum		39.600	12.565	58.374	18.500	12.562	58.374
Minimum		1.300	1.793	14.507	0.500	3.529	14.507
Std. Dev.		11.953	3.112	10.446	5.570	2.562	10.446
Brazil	Mean	19.174	5.898	38.074	12.452	5.581	38.074
	Maximum	25.400	7.908	47.000	18.500	7.528	47.000
	Minimum	13.100	3.796	25.000	7.000	3.529	25.000
	Std. Dev.	3.762	1.266	6.805	3.526	1.261	6.805
China	Mean	3.703	5.404	40.815	2.648	6.543	40.815
	Maximum	6.500	6.992	52.000	5.900	7.594	52.000
	Minimum	1.700	3.421	43.000	0.800	4.867	43.000
	Std. Dev.	1.481	1.049	6.873	1.557	0.806	6.873
India	Mean	2.874	3.243	32.778	1.326	5.576	32.778
	Maximum	5.100	5.534	46.000	2.700	7.250	46.000
	Minimum	1.300	1.793	15.000	0.500	3.620	15.000
	Std. Dev.	1.127	1.058	11.453	0.679	1.083	11.453
Russia	Mean	25.085	11.731	45.926	13.233	11.668	45.926
	Maximum	26.900	12.565	55.000	18.100	12.562	55.000
	Minimum	23.500	9.401	24.000	9.200	9.974	24.000
	Std. Dev.	1.013	1.037	8.176	2.714	0.865	8.176
South Africa	Mean	31.974	8.391	48.556	9.181	8.787	48.556
	Maximum	39.600	10.037	58.000	15.400	10.451	58.000
	Minimum	24.200	6.378	29.000	4.500	6.601	29.000
	Std. Dev.	4.723	1.102	10.146	3.318	1.175	10.146

**Source:** Authors own elaboration based on descriptive analysis. ^*^males or females with BMIs of 30 kg/m^2^ or higher as a percentage of the male/female population aged 18+ (%). ^**^mean years of schooling (years). ^***^Economic globalization index [takes value between 1 and 100 (higher values reflect higher economic globalization level)].

The causal and cointegration interactions of educational attainment, economic globalization, and obesity are, respectively, investigated with the Dumitrescu and Hurlin (42) causality test and Westerlund and Edgerton (43) LM (Lagrange Multiplier) bootstrap cointegration test in view of the fact that there exists heterogeneity and cross-sectional dependence among education, globalization, and obesity.

Cointegration tests investigate whether the long-run linear relationship among two or more series stationary even if there is not the linear relationship in the short-run (44). Therefore, cointegration test is employed to analyze the cointegration among educational attainment, economic globalization, and obesity, because increasing the educational attainment generally is a long-term phenomenon. However, causality analysis is also utilized to see the short run interaction among educational attainment, economic globalization, and obesity.

The LM bootstrap cointegration test permits autocorrelation and heteroscedasticity in the cointegration equation and also produces relatively more robust results for small sample sizes. The test is based on the LM test of McCoskey and Kao (45), and bootstrap critical values are taken into account in case there exists cross-sectional dependence (46). The cointegration test is generated from Equation (1):


(1) $$\begin{array}{l} y_{it}=\alpha_{i}+x_{it}^{{}^{\prime }}\beta_{it}+z_{it} \end{array}$$

t = 1….,T and i = 1….,N respectively specifies the time series and cross-sections and $z_{it}\;\left(z_{it}=\mu_{it}+v_{it}\overset{t}{\underset{j=1}{\sum }}n_{ij}\right)$is the error term. *n*_*ij*_ is an error term with a zero mean and $\sigma_{i}^{2}$ variance.

The null hypothesis of the cointegration test suggests a significant cointegration among education, globalization, and obesity in all countries and is tested by the LM test statistic in Equation (2).


(2) $$\begin{array}{l} LM_{N}^{+}=\frac{1}{NT^{2}}\overset{N}{\underset{i=1}{\sum }}\overset{t}{\underset{t=1}{\sum }}{\hat{\omega }}_{i}^{-2}s_{it}^{2} \end{array}$$

$s_{it}^{2}$ is partial sum of *z*_*it*_, and ${\hat{\omega }}_{i}^{-2}$ is long-term variance of μ_*it*_.

The causality analysis investigates a bidirectional interaction among educational attainment, economic globalization, and obesity. In other words, it tests whether educational attainment has a significant effect on obesity or obesity has a significant effect on educational attainment. The Dumitrescu and Hurlin (42) causality test can be utilized in case of unbalanced panels, existence of cross-sectional dependence, N > T, and T > N and the test employs the following equation:


(3) $$\begin{array}{l} y_{i,t}=\alpha_{i,t}\overset{K}{\underset{k=1}{\sum }}\gamma_{i}^{\left(k\right)}y_{i,t-k}+\overset{K}{\underset{k=1}{\sum }}\beta_{i}^{\left(k\right)}x_{i,t-k}+\varepsilon_{i,t} \end{array}$$

In (3) numbered equation, k is lag length, γ and β are respectively dependent and independent variables lags' coefficients. All variables used in the causality analysis should be stationary. The null hypothesis of the test suggests an insignificant causality between two series and the null hypothesis is tested by Wald $\left(W_{N,T}^{Hnc\;\left(Homogeneous\;non\;causality\right)}\right)$ and $Z_{N,T}^{Hnc}$ test statistics as following [see Dumitrescu and Hurlin (42) for detailed information about calculation of test statistics]:


(4) $$\begin{array}{l} W_{N,T}^{Hnc\;}=\frac{1}{N}\overset{N}{\underset{i=1}{\sum }}W_{i,T} \end{array}$$

Zhnc$\left(Z_{NT}^{Hnc}\right)$ test statistic in Equation (4) with asymptotic distribution is taken into account if *N*<*T*, but Ztild $\left(Z_{N}^{Hnc}\right)$ test statistic in Equation (5) with semi- asymptotic distribution is taken into account if *T*<*N*.


(5) $$\begin{array}{lll} Z_{NT}^{Hnc} & = & \sqrt{\frac{N}{2K}}\left(W_{N,T}^{Hnc}-K\right) \end{array}$$

(6) $$Z_{N}^{Hnc}\;=\frac{\sqrt{N\left[W_{N,T}^{Hnc}-N^{-1}\sum_{i=1}^{N}E\left(W_{i,T}\right)\right]}}{\sqrt{N^{-1}\sum_{i=1}^{N}Var\left(W_{i,T}\right)}}$$

## 4. Results and discussion

The interaction of educational attainment, economic globalization, and obesity is analyzed by cointegration and causality tests. In this context, pretests of cross-sectional dependence and heterogeneity are, respectively, investigated by LM and delta tilde tests at first. The existence of cross-sectional dependence among countries is examined with *LM*_*adj*._, LM CD, and LM tests and their results are depicted in Table 4. The alternative hypothesis of three tests (“there exists cross-sectional dependence”) is accepted because the probability values of these tests are lower than 0.05. Then, the existence of slope coefficients' homogeneity is controlled by delta tilde tests, and their results are depicted in Table 4. The alternative hypothesis of two tests (“there exists heterogeneity”) is accepted because the probability values of these tests are lower than 0.05. So, the effect of educational attainment and economic globalization on obesity in adult females and males differs among the countries.

**Table 4:** Results of cross-sectional dependence and heterogeneity tests.

**Test**	**Model 1 (Females)**		**Model 2 (Males)**	
	**Test statistic**	***P*** **value**	**Test statistic**	***P*** **value**
**Cross-sectional dependence tests**				
LM _adj_ (47)	***22.61***	***0.0000***	***27.4***	***0.000***
LM CD (48)	***3.929***	***0.0001***	***6.286***	***0.000***
LM (49)	***47.96***	***0.0000***	***55.81***	***0.000***
**Heterogeneity tests**				
Delta tilde (50)	***21.041***	***0.000***	***12.634***	***0.000***
Adjusted delta tilde (50)	***22.797***	***0.000***	***13.689***	***0.008***

**Source:** Authors own elaboration based on cross-sectional and homogeneity tests. The significant results are shown in bold and italic.

The stationarity analysis of OBS, EDU, and EG in Model 1 and Model 2 is implemented by Pesaran (46) cross-sectional augmented Dickey–Fuller (CADF) unit root test, and the results are depicted in Table 5. All series are not stationary at their level values, but the series have become stationary at first-differenced values.

**Table 5:** CADF unit root test results.

**Variables**	**Model 1 (Females)**		**Model 2 (Males)**	
	**Constant**	**Constant** + **Trend**	**Constant**	**Constant** + **Trend**
OBS	–1.178	–1.325	–0.983	–1.023
D(OBS)	–8.312^***^	**−8.793** ^***^	–6.462^***^	**−7.063** ^***^
EDU	–1.205	–1.467	–1.142	–1.156
D(EDU)	–9.735^***^	**−10.042** ^***^	–8.606^***^	**−8.993** ^***^
EG	–0.821	0.820	–0.821	0.820
D(EG)	–2.532^***^	–4.562^***^	–2.532^***^	**−4.562** ^***^

**Source:** Authors own elaboration based on unit root test. ^***^significant at 1% level. The significant results are shown in bold and italic.

The long-term interaction of educational attainment, economic globalization, and obesity in adult females and males in BRICS economies is investigated by the LM bootstrap cointegration test in deference to small sample sizes and subsistence of cross-sectional dependence. The results of the LM bootstrap cointegration test are depicted in Table 6. As a result, the null hypothesis (“there exists a significant cointegration interaction among educational attainment, economic globalization, and obesity for females and females”) is accepted, and a significant long-term relationship between the three variables is reached.

**Table 6:** LM bootstrap cointegration test.

**Models**	**Constant**			**Constant** + **Trend**		
	**Test statistic**	**Asymptotic** ***P*** **value**	**Bootstrap** ***P*** **value**	**Test statistic**	**Asymptotic** ***P*** **value**	**Bootstrap** ***P*** **value**
Model 1 (Females)	8.914	0.214	0.289	9.127	0.302	0.326
Model 2 (Males)	7.265	0.145	0.168	8.902	0.210	0.311

**Source:** Authors own elaboration based on cointegration test. Bootstrap probability values are generated using 10,000 simulations. Asymptotic *P* values are procured from standard normal distribution. Lag and lead values are 1.

The cointegration coefficients are predicted by AMG estimator (51, 52), and the coefficients are denoted in Table 7. The estimated coefficients reveal that educational attainment has a negative impact on obesity in females and males in all BRICS economies, but the negative influence of educational attainment on obesity is found to be relatively higher in females than males. Furthermore, the negative impact of educational attainment on obesity in both females and males is relatively higher in China, India, and Russia.

**Table 7:** Cointegration coefficient estimation.

**Countries**	**Model 1 (Females)**		**Model 2 (Males)**	
	**EDU**	**EG**	**EDU**	**EG**
Brazil	**−0.121** ^***^	0.007	**−0.081** ^***^	0.005
*China*	**−0.196** ^***^	**−0.023** ^***^	**−0.142** ^***^	**−0.021** ^***^
India	**−0.162** ^*^	**−0.031** ^*^	**−0.151** ^**^	**−0.011** ^**^
Russia	**−0.147** ^***^	**0.005** ^***^	**−0.144** ^***^	−0.002
South Africa	**−0.113** ^*^	**0.027** ^***^	**−0.089** ^**^	**0.012** ^***^
Panel	−0.012	−0.003	−0.0621	−0.008

**Source:** Authors own elaboration based on AMG estimation. ^***^, ^**^, and ^*^are significant at the 1, 5, and 10% levels, respectively. The significant estimations are shown in bold and italic.

On the other hand, economic globalization has a negative influence on obesity in China and India, but a positive influence on obesity in females in Russia and South Africa. Economic globalization also has a negative influence on obesity in males in China and India, but a positive influence on obesity in males in South Africa.

The findings of the study are compatible with theoretical expectations and related empirical literature about the education–obesity nexus. The cointegration analysis indicates that improvements in educational attainment contribute to decreases in obesity in the long run. Educational attainment proxied by different indicators can make a direct contribution to decreases in obesity by raising awareness of obesity-related health problems and encouraging healthy eating and regular physical activity. Higher educational attainment can also cause individuals to earn higher income and, in turn, foster healthy nutrition and lifestyles. Furthermore, educational attainment can contribute to decreases in obesity by enhancing economic growth and development because educational attainment is a critical factor for human capital, which is a significant determinant of economic growth and development. In the related empirical literature, Devaux et al. (15), Anyanwu et al. (23), Faeh et al. (24), Brunello et al. (25), Chung et al. (26), Chung and Lim (27), Hsieh et al. (28), Witkam et al. (29), Iriyani et al. (30), and Monteiro et al. (22) also discovered a negative influence of various education indicators on obesity in different countries with different income levels in a similar way.

Our findings also reveal that the influence of educational attainment on obesity is relatively higher in females than males in the BRICS economies. Witkam et al. (29) and Monteiro et al. (22) similarly reached the conclusion that education is more effective for obesity in women than men. Furthermore, the influence of educational attainment on obesity in both genders varies among the BRICS economies. China and India achieved significant progress in educational attainment, GDP per capita and human development during the 1990–2016 period and Russia was the leading country among the BRICS economies in terms of socio-economic development as seen in Table 8. Therefore, we evaluate that the variations about the influence of educational attainment on obesity can be resulted from the differences in human and economic development of the BRICS economies.

**Table 8:** Real GDP per capita and human development index in BRICS economies.

**Countries**	**GDP per capita** **(constant 2015** **US $)**		**Human development** **index**	
	**1990**	**2016**	**1990**	**2016**
Brazil	6,155.645	8,455.312	0.61	0.755
China	905.031	8,516.514	0.484	0.74
India	532.755	1,719.318	0.434	0.639
Russian Federation	7,849.512	9,313.967	0.743	0.828
South Africa	5,031.464	6,209.365	0.632	0.719

**Source:** UNDP (40) and World Bank (53).

Economic globalization can influence obesity through fostering economic growth and development, increasing accessibility to obesogenic products, disseminating the modern workplace, technology use, and motorized transportation, and urbanization (17, 18). Therefore, which of these factors is dominant determines the effect of economic globalization on obesity. In the related empirical literature, García (17), De Vogli et al. (32), Goryakin et al. (33), Costa-Font and Mas (34), Lopez et al. (36), Fox et al. (38), and Lin et al. (37) discovered a positive influence of various globalization components on obesity. Only Ghosh (35) reached the conclusion that the influence of globalization on obesity varies based on countries' income levels. In the study, the influence of economic globalization on obesity differs among the BRICS economies and the coefficients are revealed to much lower when compared with educational attainment. The positive influence of economic globalization on obesity in females in Russia and South Africa and in males in South Africa is consistent with the related empirical literature to a great extent. However, economic globalization has a very small negative influence on obesity in both genders in China and India in compatible with findings by Ghosh (35). The weak positive or negative influence of economic globalization on obesity can be probably resulted from low economic globalization levels of BRICS economies, because the mean of economic globalization in the BRICS economic is only 41.203 over the 1990–2016 period. Furthermore, the mean of economic globalization in South Africa, Russia, China, Brazil, and India for the 1990–2016 period are respectively 48.556, 45.926, 40.815, 38.074, and 32.778.

The causal interaction of educational attainment, economic globalization, and obesity for females and males is investigated by the Dumitrescu and Hurlin (42) causality test, and the results of this test are depicted in Table 9. The causality analysis uncovered a unidirectional causality from educational attainment and economic globalization to obesity for both genders. In other words, economic globalization and educational attainment have a significant effect on obesity in the short term. A significant interaction between educational attainment, economic globalization, and obesity is theoretically expected, but the studies have mainly conducted one-way analyses from educational attainment and economic globalization to obesity and discovered a significant influence of both variables on obesity. In this research, mutual interaction of educational attainment, economic globalization, and obesity is analyzed, but an insignificant influence from obesity on educational attainment and economic globalization is discovered. Therefore, the causality findings are revealed to be compatible with the related empirical literature to a great extent.

**Table 9:** Causality analysis findings.

		**Females**		**Males**	
**Null hypothesis**	**Test**	**Test statistics**	***P*** **value**	**Test statistics**	***P*** **value**
***DEDU** **↛DOBS***	***Whnc***	***5.721***	***0.000***	***8.453***	***0.000***
	***Zhnc***	***6.909***	***0.000***	***8.912***	***0.000***
	***Ztild***	***7.112***	***0.000***	***9.067***	***0.000***
DOBS ↛DEDU	*Whnc*	1.256	0.109	1.563	0.109
	*Zhnc*	1.310	0.115	1.879	0.127
	*Ztild*	1.378	0.128	2.112	0.156
***DEG** **↛DOBS***	***Whnc***	***6.808***	***0.006***	***5.302***	***0.000***
	***Zhnc***	***7.114***	***0.000***	***6.478***	***0.000***
	***Ztild***	***7.913***	***0.001***	***6.801***	***0.012***
DOBS ↛DEG	*Whnc*	1.195	0.132	0.982	0.145
	*Zhnc*	1.624	0.149	1.314	0.166
	*Ztild*	2.089	0.161	1.722	0.210

**Source:** Authors own elaboration based on causality test. The significant results are shown in bold and italic.

## 5. Conclusion

Worldwide obesity has increased considerably, and obesity is accepted as a global epidemic and one of the most important threats to public health. Obesity is not only a significant source of many NCDs but also leads many negative economic and social implications for societies. Therefore, international institutions and national governments have tried to control and decrease abnormal increases in obesity.

In this study, the influence of educational attainment and economic globalization on obesity in adult females and males are separately investigated in sample of BRICS economies through causality and cointegration analyses by paying attention to significant differences in obesity rate between females and males. The causality analysis reveals that education and economic globalization have significant influence on female and male obesity in the short term. On the other hand, the cointegration analysis shows that educational attainment has a negative influence on obesity in both adult females and males, but the influence of educational attainment on obesity is generally revealed to be higher in females than males. Furthermore, the influence of economic globalization on obesity varies among the BRICS economies.

Our findings and the related literature indicate that educational attainment has a negative influence on obesity in countries with different income levels and also suggest that educational attainment is one of the most effective instruments for decreasing obesity. Therefore, increasing educational attainment should be used as a policy instrument to decrease obesity. In addition, the related literature has widely revealed a positive influence of economic globalization on obesity because economic globalization can increase access to obesogenic products and disseminate the modern workplace, technology use, motorized transportation, urbanization, and cultural changes. However, a positive influence of economic globalization on obesity is discovered for females in Russia and South Africa and for males in South Africa and a very small negative influence of economic globalization on obesity is revealed for both genders in China and India. Both positive and negative influence of economic globalization on obesity is very small when compared with that of educational attainment. We evaluate that the small influence of economic globalization on obesity can be resulted low economic globalization levels of the BRICS economies.

## Data availability statement

The original contributions presented in the study are included in the article/supplementary material, further inquiries can be directed to the corresponding author.

## Author contributions

All authors listed have made a substantial, direct, and intellectual contribution to the work and approved it for publication.
